# External Validation of the MRI-DRAGON Score: Early Prediction of Stroke Outcome after Intravenous Thrombolysis

**DOI:** 10.1371/journal.pone.0099164

**Published:** 2014-06-04

**Authors:** Guillaume Turc, Pierre Aguettaz, Nelly Ponchelle-Dequatre, Hilde Hénon, Olivier Naggara, Xavier Leclerc, Charlotte Cordonnier, Didier Leys, Jean-Louis Mas, Catherine Oppenheim

**Affiliations:** 1 Department of Neurology, Hôpital Sainte-Anne, Paris, France & Université Paris Descartes, Sorbonne Paris Cité, INSERM UMR S894; 2 Department of Radiology, Hôpital Sainte-Anne, Paris, France & Université Paris Descartes, Sorbonne Paris Cité, INSERM UMR S894; 3 Department of Neurology, Lille University Hospital, Lille, France & Université Lille Nord de France, UDSL, EA 1046; 4 Department of Radiology, Lille University Hospital, Lille, France & Université Lille Nord de France, UDSL, EA 1046; University Hospital-Eppendorf, Germany

## Abstract

**Background and Purpose:**

The aim of our study was to validate in an independent cohort the MRI-DRAGON score, an adaptation of the (CT-) DRAGON score to predict 3-month outcome in acute ischemic stroke patients undergoing MRI before intravenous thrombolysis (IV-tPA).

**Methods:**

We reviewed consecutive (2009–2013) anterior circulation stroke patients treated within 4.5 hours by IV-tPA in the Lille stroke unit (France), where MRI is the first-line pretherapeutic work-up. We assessed the discrimination and calibration of the MRI-DRAGON score to predict poor 3-month outcome, defined as modified Rankin Score >2, using c-statistic and the Hosmer-Lemeshow test, respectively.

**Results:**

We included 230 patients (mean ±SD age 70.4±16.0 years, median [IQR] baseline NIHSS 8 [5]–[14]; poor outcome in 78(34%) patients). The c-statistic was 0.81 (95%CI 0.75–0.87), and the Hosmer-Lemeshow test was not significant (p = 0.54).

**Conclusions:**

The MRI-DRAGON score showed good prognostic performance in the external validation cohort. It could therefore be used to inform the patient's relatives about long-term prognosis and help to identify poor responders to IV-tPA alone, who may be candidates for additional therapeutic strategies, if they are otherwise eligible for such procedures based on the institutional criteria.

## Introduction

Intravenous thrombolysis (IV-tPA) is the only licensed treatment for acute ischemic stroke. However, about half of the treated patients will not achieve functional independence at 3 months, mostly due to delayed or lack of recanalization [1]–[2]. Therefore, it would be useful to quickly identify patients unlikely to respond to IV-tPA, who may be candidates for complementary treatment strategies, such as endovascular therapy. The DRAGON score (“CT-DRAGON”), which incorporates clinical and radiological parameters easily available at admission, has shown good performance to predict 3-month poor outcome after IV-tPA [3]–[4]. We have recently adapted this score for anterior circulation stroke patients undergoing MRI as the first-line diagnostic tool (MRI-DRAGON score, Table 1), and performed an internal cross-validation [5]. Our aim was to perform the first validation of this score in an independent population.

**Table 1:** The MRI-DRAGON Score (0–10 points).

Parameters (before IV-tPA)	Points
	
**M**1 occlusion	1
**D**WI-ASPECTS ≤5	1
Prestroke m**R**S >1	1
**A**ge	
65–79	1
≥80	2
**G**lucose level >8 mmol/L	1
**O**nset-to-treatment time >90 min	1
**N**IHSS score	
5–9	1
10–15	2
>15	3

IV-tPA: intravenous thrombolysis; M1: M1 segment of the middle cerebral artery. Any proximal M1 occlusion on admission MR Angiography was considered, irrespective of the status of other arteries.

mRS: Modified Rankin scale. NIHSS: National Institute of Health Stroke Scale. DWI-ASPECTS: Diffusion-weighted imaging Alberta Stroke Programme Early CT score.

## Methods

### Study setting

The validation cohort consisted of consecutive patients included in the Lille University Hospital registry of strokes treated with IV-tPA between May 2009 and August 2013. Since May 2009, MRI has been the first-line pretherapeutic imaging modality in candidates for thrombolysis in this center. Inclusion criteria were: (a) anterior circulation stroke, (b) prestroke modified Rankin Scale (mRS) score ≤2, (c) MRI at baseline, (d) treatment exclusively by IV-tPA within 4.5 hours after stroke onset. Clinical variables of the MRI-DRAGON model were prospectively collected. Poor outcome was defined as 3-month mRS >2 [3], [5]. Miserable outcome was defined as 3-month mRS >4.

### Imaging

Pretreatment MRI (1.5-T Philips Achieva) included diffusion-weighted imaging (DWI), FLAIR, T2* and intracranial time-of-flight MR angiography (MRA). The imaging parameters of the MRI-DRAGON score were retrospectively assessed by neuroradiologists (P.A., O.N., C.O., X.L.) blinded to clinical data. Any proximal (M1) middle cerebral artery (MCA) occlusion on admission MRA was considered, irrespective of the status of the other arteries. DWI-ASPECTS was scored on pretreatment MRI and dichotomized into >5 *vs.* ≤5 (primary analysis) [5]–[6]. However, because the best DWI-ASPECTS cut-off for poor outcome prediction is currently debated [5], [7], [8], we performed a sensitivity analysis using the ≤7 DWI-ASPECTS cut-off instead of ≤5.

### Statistical analysis

The derivation of the MRI-DRAGON score has been described in the original publication [5]. In the present study, continuous variables with a normal distribution were described as mean ± standard deviation (SD) and non-normally distributed variables were described as median and interquartile range (IQR). Univariate and multivariate binary logistic regression analysis was performed with poor outcome (or miserable outcome in supplemental analysis) as the dependent variable. All variables of the MRI-DRAGON score were included in the multivariate model. Discrimination of the MRI-DRAGON score was assessed using c-statistic (*i.e.* Area Under the ROC curve) with 95% confidence interval. Calibration was assessed using the Hosmer-Lemeshow test to check for significant differences between the observed and predicted risks of poor outcome [9]. Statistical analysis was performed using SAS 9.3 (SAS institute, Cary, NC).

### Ethics statement

The stroke registry of the present study was declared at the institutional data protection board (“Comité consultatif sur le traitement de l'information en matière de recherche dans le domaine de la santé”). In accordance with the French legislation, the study did not need approval by an Ethics Committee, because it implied only retrospective analysis of anonymized data collected prospectively as part of routine clinical care. The study has been performed in accordance with the ethical standards laid down in the 1964 declaration of Helsinki and its later amendments. Patients or their relatives gave a verbal informed consent (documented within the patient's medical record) for the inclusion of their data in a registry, in accordance with the French law concerning research in Human.

## Results

Among 479 patients included in the registry during the study period, 230 fulfilled the inclusion criteria (Table 2); 249 patients were not eligible because of CT scanner as admission imaging (n = 65), prestroke mRS >2 (n = 49), unknown or >4.5 hours onset-to-treatment time (OTT) (n = 46), posterior circulation stroke (n = 42), additional endovascular therapy (n = 20), missing data (n = 17), incomplete MRI protocol (n = 8), or stroke mimics (n = 2). Patients not eligible because they underwent CT scan or bridging therapy, had significantly higher baseline NIHSS scores than included patients (p<0.001). When compared to the derivation cohort [5], patients in this validation cohort were significantly older, had lower admission NIHSS scores, lower rates of M1 occlusion, and better outcome (Table 2). In univariate analysis, all parameters of the MRI-DRAGON score, except OTT, were significantly associated with poor outcome (Table 3). In multivariate analysis, NIHSS score on admission and age remained significantly associated with poor outcome.

**Table 2:** Characteristics of the Validation and Derivation[5] cohorts.

	Validation Cohort (Lille)	Derivation Cohort (Sainte-Anne) n = 228	p-value
	n = 230		
***Baseline characteristics***			
Male gender	104 (45)	122 (54)	0.08
Age, mean ±SD, years	70.4±16.0	67.3±14.9	0.03
Hypertension	156 (68)	124 (54)	0.003
Diabetes mellitus	38 (16)	30 (13)	0.31
Current smoking	43 (19)	42 (18)	0.94
Atrial fibrillation	48 (21)	63 (28)	0.09
Previous stroke	20 (9)	20 (9)	0.98
Prestroke mRS >1	23 (10)	6 (3)	0.001
			
***Before IV-tPA***			
NIHSS, median (IQR)	8 (5–14)	14 (8–19)	<0.0001
OTT, median (IQR), min	149 (120–191)	160 (125–195)	0.26
Serum glucose, mean ±SD, mmol/L	7.3±2.7	6.7±2.2	0.01
Systolic BP, mean ±SD, mmHg	156.3±20.2	152.8±22.9	0.08
Diastolic BP, mean ±SD, mmHg	82.5±14.1	82.9±15.6	0.94
M1 occlusion	75 (33)	135 (59)	<0.0001
DWI-ASPECTS, median (IQR)	8 (7–9)	7 (6–8)	<0.0001
DWI-ASPECTS ≤5	33 (14)	46 (20)	0.10
			
***Outcome***			
3-month mRS >2	78 (34)	98 (43)	0.05
3-month mRS >4	23 (10)	42 (18)	0.01

Numbers in parentheses are percentages, unless indicated. SD: standard deviation; IQR: interquartile range; IV-tPA: intravenous thrombolysis; OTT: Onset-to-treatment time; M1: M1 segment of the middle cerebral artery; mRS: Modified Rankin scale. NIHSS: National Institute of Health Stroke Scale. DWI-ASPECTS: Diffusion-weighted imaging Alberta Stroke Programme Early CT score.

**Table 3:** Association between MRI-DRAGON parameters and poor outcome at 3 month (n = 230).

	mRS >2	mRS ≤2	Univariate analysis Multivariate analysis			
	N = 78	N = 152				
	n (%)	n (%)	OR (95% CI)	p-value	*Adjusted* OR (95% CI)	p-value
***MRI-DRAGON parameters***						
**M**1 occlusion						
Yes	43 (55)	32 (21)	4.61 (2.55–8.33)	<0.0001	1.58 (0.71–3.52)	0.26
No	35 (45)	120 (79)	1.00			
**D**WI-ASPECTS						
≤5	19 (24)	14 (9)	3.17 (1.49–6.75)	0.003	1.14 (0.42–3.12)	0.80
>5	59 (76)	138 (91)	1.00			
Prestroke m**R**S>1						
Yes	13 (17)	10 (7)	2.84 (1.18–6.81)	0.02	1.93 (0.63–5.92)	0.25
No	65 (83)	142 (93)	1.00			
**A**ge						
≥80 years	42 (54)	39 (26)	4.16 (2.04–8.52)	0.0001	3.01 (1.25–7.29)	0.02
65–79 years	21 (27)	55 (36)	1.48 (0.69–3.15)		1.23 (0.49–3.07)	
<65 years	15 (19)	58 (38)	1.00		1.00	
**G**lucose level						
>8 mmol/L	25 (32)	28 (18)	2.09 (1.12–3.91)	0.02	1.83 (0.84–3.97)	0.13
≤8 mmol/L	53 (68)	124 (82)	1.00			
**O**nset-to-treatment time						
>90 min	73 (94)	140 (92)	1.25 (0.43–3.69)	0.68	1.45 (0.39–5.46)	0.58
≤90 min	5 (6)	12 (8)	1.00			
**N**IHSS score on admission			1.22 (1.15–1.30)*	<0.0001*	1.19 (1.11–1.28)*	<0.0001*
>15	36 (46)	11 (7)	-		-	
10–15	20 (26)	32 (21)	-		-	
5–9	13 (17)	61 (40)	-		-	
0–4	9 (11)	48 (32)	-		-	

All variables included in the multivariate model are presented in the table. M1  =  M1 segment of the middle cerebral artery. * per 1-point NIHSS score increase.

mRS: Modified Rankin scale. NIHSS: National Institute of Health Stroke Scale. DWI-ASPECTS: Diffusion-weighted imaging Alberta Stroke Programme Early CT score.

**Figure 1:**
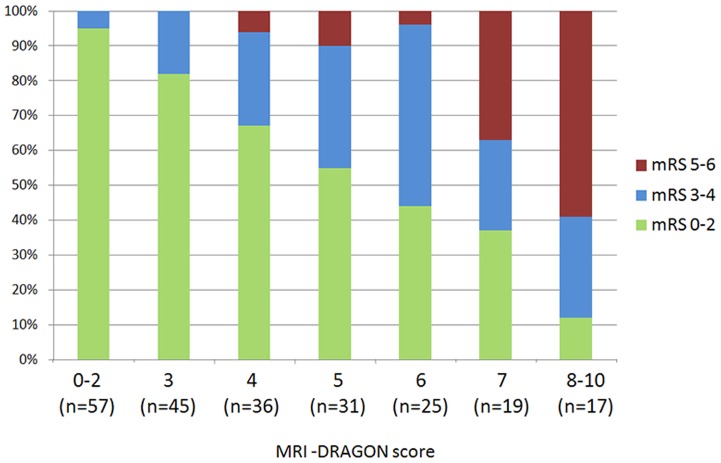
Three-month outcome according to MRI-DRAGON score. mRS: modified Rankin Scale.

The distribution of the 3-month outcomes per increasing point of MRI-DRAGON score is shown in Figure 1. The c-statistic for poor outcome prediction was 0.81 (95%CI = 0.75–0.87). The Hosmer-Lemeshow test showed no significant difference between the observed and predicted risks of poor outcome (Chi-square = 7.0, p = 0.54). Using the ≤7 DWI-ASPECTS cut-off in sensitivity analysis led to similar results (c-statistic = 0.81, 95%CI 0.76–0.87). In supplemental analysis, the c-statistic of the MRI-DRAGON score (using the DWI-ASPECTS ≤5 cut-off) to predict miserable outcome was 0.89 (95%CI 0.84–0.95); the Hosmer-Lemeshow test was non-significant (p = 0.52).

## Discussion

The ability of the MRI-DRAGON score to predict poor outcome was good (c-statistic = 0.81) and similar to that reported in the derivation cohort (0.83) or in the CT-DRAGON score publications (0.79 to 0.85) [3]–[5], [10], [11]. The DRAGON score can quickly be assessed by a stroke specialist at admission, is straightforward, and cost-free. It could help to inform relatives about the patient's 3-month prognosis. In addition, by identifying poor responders to IV-tPA, the CT- or MRI-DRAGON score could be used, irrespective of the imaging screening method, to select patients for future therapeutic trials or to help clinical decision-making in patients who cannot be included in a randomized trial of bridging therapy [4]. Importantly, despite a high probability of poor outcome according to the MRI-DRAGON score, there is currently no rational to withhold IV-tPA if indicated.

While the derivation cohort was similar to the Safe Implementation of Thrombolysis (SITS) registry's data regarding age, stroke severity and outcome, patients in the validation cohort had less severe strokes, consistent with a lower occurrence of M1 occlusion [1]. We acknowledge that the validation population was potentially selected, since patients imaged by MRI had less severe strokes than those imaged by CT, and 18/20 (90%) patients who underwent additional endovascular therapy had an M1 occlusion. However, despite differences in stroke severity in the derivation and validation cohort, the MRI-DRAGON score showed good prognostic performances in both settings, which could strengthen the generalizability of our results [9].

The monocenter design may raise some concerns. However, our score is an adaptation of the widely validated CT-DRAGON score, rather than a novel prognostic tool [3], [4], [10], [11]. Besides, several parameters of the MRI-DRAGON score, notably MRI-based parameters, did not reach significance in multivariate analysis in the validation cohort. Yet, M1 occlusion and DWI-ASPECTS have been shown by others to be independent predictors of outcome[12], [13] and were significantly associated with poor outcome in our univariate analysis. We may have lacked statistical power for these parameters, given the low prevalence of M1 occlusion and low DWI-ASPECTS in the validation cohort. Although our main multivariate logistic model (poor outcome prediction) is unlikely to be overfitted [14], [15], this concern remains for the supplemental analysis, given the small number of patients who experienced miserable outcome. Therefore, the c-statistic of the MRI-DRAGON score to predict miserable outcome might be overestimated in our study, and should be interpreted with caution. Finally, as we included only anterior circulation strokes, our findings cannot apply to posterior circulation stroke patients. Further data would be needed to determine if our score can be adapted for posterior circulation stroke prediction, for example using the Posterior DWI ASPECTS score [16].

In conclusion, the MRI-DRAGON score showed good prognostic performance in the external validation cohort. It could help identifying patients unlikely to respond to IV-tPA alone and may be candidates for additional therapeutic strategies, if they are otherwise eligible for such procedures based on the institutional criteria.
